# The Capital Cost of London and Provincial Hospitals

**Published:** 1911-10-21

**Authors:** Claude Wright

**Affiliations:** General Secretary Dr. Barnardo's Homes.


*To the Editor of* The Hospital.

### THE CAPITAL COST OF LONDON AND PROVINCIAL HOSPITALS.

*To the Editor of* The Hospital.

Sir,—I wonder whether you could tell me what is a fair average cost on capital account per bed in London and provincial hospitals? That is to say, what do the buildings work out at per bed? I, of course, know what is the cost per annum for upkeep, but I want to get the cost per head on capital account.

For any information you can give me I shall be much obliged.—Yours faithfully,

October 10. Claude Wright, General Secretary

Dr. Barnardo's Homes.

[Presuming Mr. Wright means the cost per bed as represented by the actual expenditure in the whole of a modern hospital, we could mention instances where the total expenditure represents a cost of something like £1,000 per bed. In our judgment, the present cost of hospital buildings is infinitely greater than it ought to be and need be. In our view the average cost should seldom if ever exceed a sum of from £200 to £300 per bed.—Ed. The Hospital.]

